# The Hidden Genomic Diversity, Specialized Metabolite Capacity, and Revised Taxonomy of *Burkholderia* Sensu Lato

**DOI:** 10.3389/fmicb.2021.726847

**Published:** 2021-09-22

**Authors:** Alex J. Mullins, Eshwar Mahenthiralingam

**Affiliations:** Microbiomes, Microbes and Informatics Group, Organisms and Environment Division, School of Biosciences, Cardiff University, Cardiff, United Kingdom

**Keywords:** *Burkholderia* sensu lato, phylogenomics, taxonomy, average nucleotide identity, biosynthetic gene clusters, specialized metabolites

## Abstract

*Burkholderia* sensu lato is a collection of closely related genera within the family Burkholderiaceae that includes species of environmental, industrial, biotechnological, and clinical importance. Multiple species within the complex are the source of diverse specialized metabolites, many of which have been identified through genome mining of their biosynthetic gene clusters (BGCs). However, the full, true genomic diversity of these species and genera, and their biosynthetic capacity have not been investigated. This study sought to cluster and classify over 4000 *Burkholderia* sensu lato genome assemblies into distinct genomic taxa representing named and uncharacterized species. We delineated 235 species groups by average nucleotide identity analyses that formed seven distinct phylogenomic clades, representing the genera of *Burkholderia* sensu lato: *Burkholderia*, *Paraburkholderia*, *Trinickia*, *Caballeronia*, *Mycetohabitans*, *Robbsia*, and *Pararobbisa.* A total of 137 genomic taxa aligned with named species possessing a sequenced type strain, while 93 uncharacterized species groups were demarcated. The 95% ANI threshold proved capable of delineating most genomic species and was only increased to resolve several closely related species. These analyses enabled the assessment of species classifications of over 4000 genomes, and the correction of over 400 genome taxonomic assignments in public databases into existing and uncharacterized genomic species groups. These species groups were genome mined for BGCs, their specialized metabolite capacity calculated per species and genus, and the number of distinct BGCs per species estimated through kmer-based de-replication. *Mycetohabitans* species dedicated a larger proportion of their relatively small genomes to specialized metabolite biosynthesis, while *Burkholderia* species harbored more BGCs on average per genome and possessed the most distinct BGCs per species compared to the remaining genera. Exploring the hidden genomic diversity of this important multi-genus complex contributes to our understanding of their taxonomy and evolutionary relationships, and supports future efforts toward natural product discovery.

## Introduction

**FIGURE 1:**
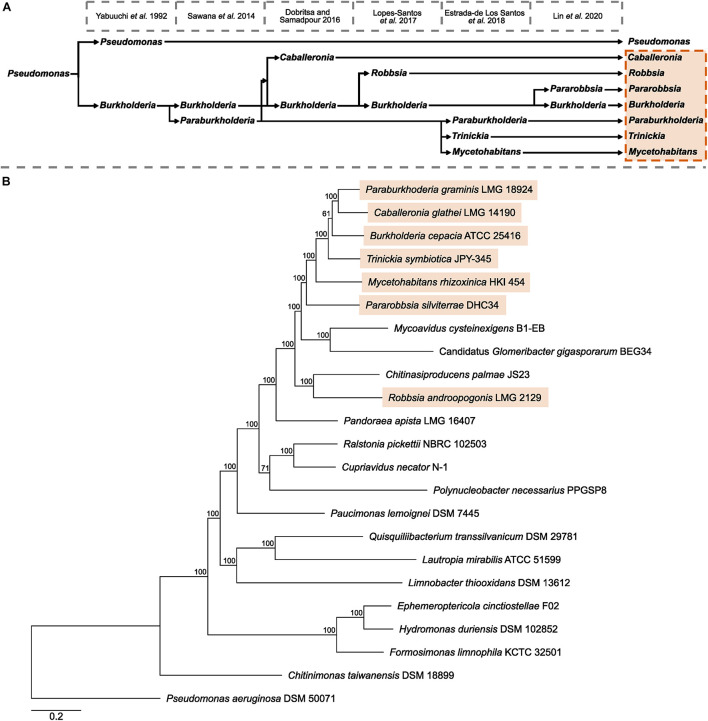
Taxonomic history of *Burkholderia* sensu lato and Burkholderiaceae phylogeny. **(A)** A timeline of key publications that define the splitting of existing genera and the formation novel genera beginning with the description of *Burkholderia* as a separate genus to *Pseudomonas* in 1992. **(B)** The phylogeny of Burkholderiaceae genera was based on 629 orthogroups with a minimum of 91.3% of genomes having single-copy genes in any orthogroup. The maximum likelihood phylogeny was constructed using the LT model determined by the RAxML automatic amino acid substitution model assignment with 100 bootstraps. The phylogeny contains type strain representatives of 21 of the 22 validly named species that, in turn, represent the genera of Burkholderiaceae, and a representative of the invalidly named “Candidatus *Glomeribacter*” genus. The phylogeny was rooted with *Pseudomonas aeruginosa* DSM 50071. Genera currently recognized as *Burkholderia* sensu lato are highlighted in orange. Scale bar represents the number of base substitutions per site.

The multi-genus complex known as *Burkholderia* sensu lato has undergone multiple expansions and refinements following the emergence and description of the genus *Burkholderia* in 1992 (Yabuuchi et al., 1992; Figure 1A). The first splitting of *Burkholderia* resulted in the recognition of a genus composed of predominantly environmental species, known as *Paraburkholderia*, and was supported by conserved sequence indels and a phylogeny of concatenated conserved proteins (Sawana et al., 2014). Subsequent novel genera *Caballeronia* (Dobritsa and Samadpour, 2016) and *Robbsia* (Lopes-Santos et al., 2017) were formed from *Burkholderia* and *Paraburkholderia* species previously recognized as sub-clades within *Paraburkholderia* and outlier species (Estrada-de Los Santos et al., 2013; Sawana et al., 2014). Amid the burgeoning publicly available whole-genome sequencing data the genus *Paraburkholderia* was divided to include the fungal-associated *Mycetohabitans*, and *Trinickia* that encompassed several nodulating species (Estrada-de los Santos et al., 2018). The most recent addition to the *Burkholderia* sensu lato was the description of the genus *Pararobbsia* (Lin et al., 2020) comprised of two species closely related to *Robbsia*.

The original gold standard for species delineation was DNA-DNA hybridisation (DDH) with a species threshold set at 70% hybridization between bacterial genomes (Goris et al., 2007; Richter and Rosselló-Móra, 2009). Taxonomic standards have since transitioned away from molecular based comparisons and embraced *in silico* methods as the gold standard for assessing genome similarity and subsequently defining species (Goris et al., 2007; Richter and Rosselló-Móra, 2009). Average nucleotide identity (ANI) analysis offers a robust and rapid means of attributing species status on newly sequenced bacterial genomes, typically using a species threshold of 95% (Richter and Rosselló-Móra, 2009; Jain et al., 2018) but can be extended above 95%, and up to 97%, to separate closely related named species (Richter and Rosselló-Móra, 2009; Ciufo et al., 2018; Palmer et al., 2020; Parks et al., 2020). The taxonomic potential of this technique has been exploited by the Genome Taxonomy Database (GTDB) in an attempt to address the considerable influx of bacterial and archaeal genome sequences (Parks et al., 2020). While similar, targeted efforts were recently applied to understand and consolidate the existing genomic sequence data of the *Burkholderia cepacia* complex, a sub-group of closely related species within *Burkholderia*, on a curated collection of 116 high-quality or taxonomically significant genomes (Jin et al., 2020).

Previous studies have used phylogenetic techniques to highlight the relationship between named taxa within *Burkholderia* sensu lato and demarcate genus boundaries (Depoorter et al., 2016; Dobritsa et al., 2017; Estrada-de los Santos et al., 2018), but fail to recognize the genomic diversity of undefined, uncharacterized species. Such analyses perform an important taxonomic service by re-defining and consolidating existing species into their correct, respective genera. However, beyond the domain-level mass classification efforts of GTDB (Parks et al., 2020), and the *Burkholderia cepacia* complex restricted genomic diversity analysis (Jin et al., 2020), no study has attempted to understand the evolutionary intricacies of genera defined by the *Burkholderia* sensu lato and their broader relationship in the family Burkholderiaceae. Given the clinical, agricultural, and industrial significance of *Burkholderia* sensu lato species, highlighting the existence of the hidden genomic diversity beyond named species would re-define our understanding of current taxonomy across multiple important genera.

*Burkholderia* species are known to produce an array of diverse specialized metabolites with properties such as cytotoxicity, antimicrobial activity, and virulence functions (Kunakom and Eustáquio, 2019). Many of these specialized metabolites are discovered at the strain level (Kunakom and Eustáquio, 2019), while a minority of natural products are characterized at the species level (Thongkongkaew et al., 2018; Mullins et al., 2019; Jones et al., 2021). A holistic understanding of the specialized metabolite biosynthetic capacity has been ventured in metabolically talented species such as *Burkholderia ambifaria* (Mullins et al., 2019) and *Burkholderia gladioli* (Jones et al., 2021), however, such in-depth analyses require sufficient genomic sequence data which are lacking for most species. In contrast to *Burkholderia*, the natural product library of other *Burkholderia* sensu lato genera is sparce, reflecting either a lack of biosynthetic potential, unsuitable genetic tools, or unknown biosynthetic gene cluster (BGC) activation conditions. Recent progress has begun to unlock the natural product capacity of *Paraburkholderia* and *Mycetohabitans* (Wang et al., 2018; Zheng et al., 2020), but many other species across *Burkholderia* sensu lato have yet to be explored for novel metabolites. There is evidence of considerable variation in specialized metabolite potential within *Burkholderia* sensu lato based on a focused investigation of 15 diverse genomes spanning several genera (Depoorter et al., 2016). Although larger genomic collections are necessary to detail the intra-species variation that is known to exist in species such as *B. ambifaria* (Mullins et al., 2019) and *B. gladioli* (Jones et al., 2021).

This study focused on exploiting the wealth of >4000 publicly available genomic sequences to define the expanded taxonomy and biosynthetic capacity of genera within the *Burkholderia* sensu lato. The relative position of *Burkholderia* sensu lato within the Burkholderiaceae was established by constructing a family level phylogeny, providing evidence of additional genera in *Burkholderia* sensu lato and identifying a potentially novel genus representative. By leveraging rapid genome clustering and comparison tools, we delineated 235 genomic species groups, including 93 uncharacterized species groups, and re-classified more than 450 genomes from a collection of over 4000 genome sequences. A multi-genus phylogeny composed of type and proxy-type strains representing named and uncharacterized species, respectively, revealed the hidden diversity of the genus complex. Finally, we calculated the biosynthetic capacity and distinct BGCs count of all 235 species groups across the seven genera, illustrating both species and genus level variations in biosynthetic potential. These analyses provide an opportunity to direct future natural product discovery efforts by systematically targeting demarcated species groups of biosynthetic interest within the *Burkholderia* sensu lato.

## Materials and Methods

### Defining Species Groups and Constructing Multi-Genus Phylogeny

**FIGURE 2:**
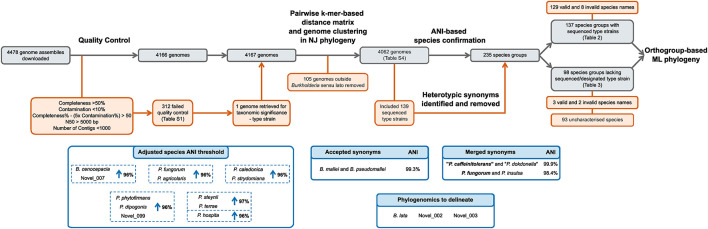
Curation and classification of genomes into species groups. A collection of 4478 genome assemblies was reduced to 4062 genomes following quality control and inclusion of taxonomically important genomes. ANI analyses delineated the 4062 genome assemblies into 235 species groups after heterotypic synonym detection, representing 137 named species groups with sequenced type strains, and 98 species groups without sequenced type strains. Species requiring ANI threshold adjustments are indicated in blue boxes.

All bioinformatic analyses were performed using the Cloud Infrastructure for Microbial Bioinformatics (CLIMB) (Connor et al., 2016). The initial download of genomic data, genome clustering, and neighbor-joining phylogeny construction was enabled by a suite of scripts, Bacsort, available at GitHub^1^. Using the wrapper download_genomes.sh script, all genomes assemblies of the genera *Burkholderia, Paraburkholderia*, *Caballeronia*, *Trinickia*, *Mycetohabitans*, *Robbsia*, and *Pararobbsia* were downloaded from NCBI (July 2020) using the ncbi-genome-download v0.2.11^2^, and kmer sketches generated with Mash v2.2.2 (Ondov et al., 2016), respectively. The genome collection was subsequently updated to include assemblies deposited up to October 2020. Genome assembly quality was assessed with CheckM v1.1.2 taxonomic-specific workflow (Parks et al., 2015). The criteria for genome quality assessment were adapted from those used in the GTDB (Parks et al., 2020) that required greater than 50% completeness, less than 10% contamination, a metric of completeness% – (5x contamination%) greater than 50, an N50 value above 5000 bp, and fewer than 1000 contigs. The Bacsort script cluster_genera.py removed genome redundancy from the dataset by clustering highly similar genomes using Mash v2.2.2 (Ondov et al., 2016), and a representative genome chosen based on highest N50 value. A pairwise distance matrix was generated from the representative genomes with the Bacsort script mash_distance_matrix.sh, and a neighbor-joining tree constructed in R v3.6.3 using the bionj_tree.R script. Clades were manually identified in the neighbor-joining tree and associated with named species. Clades that were not associated with a named species were assigned a proxy-type strain based on the genome with the highest N50 value (Parks et al., 2020). Overall, the genomic analysis started with 4478 downloaded genomic assemblies of which 4062 were subsequently analyzed (Figure 2).

Average nucleotide identity analyses were performed using fastANI v1.2 (Jain et al., 2018) with a species threshold of 95% to confirm genome identities compared to the type strain or proxy-type strain genome. A final all-vs.-all ANI analysis was performed on type and proxy-type strains with the alignment-based tool pyANI v0.2.10 (Pritchard et al., 2016) to identify heterotypic synonyms, confirm the type and proxy-type strains represented distinct species, and refine genome classification if necessary. If the ANI values between type and proxy-type strains were > 98% the two species were considered synonyms. The ANI species threshold was raised to distinguish validly named species if their respective type strains possessed 95% – 97% pairwise ANI values (Parks et al., 2020). Pairwise ANI values were considered robust if one of the asymmetric pairwise alignment fractions (AFs) were greater than 70% (Parks et al., 2020). Incidences where species groups could not be easily delineated by ANI alone, core-gene phylogenomics was performed to identify evolutionary clades. Core genes were predicted and aligned by Roary v3.13.0 (Page et al., 2015) implementing MAFFT v7.455 (Katoh and Standley, 2013), and a maximum likelihood phylogeny with 100 bootstraps constructed with RAxML v8.2.12 (Stamatakis, 2014) compiled with PTHREADS and SSE3.

Following delineation of genomes into species groups and assigning type and proxy-type strain genomes, a multi-genus phylogeny was constructed to highlight their diversity. OrthoFinder v2.4.0 (Emms and Kelly, 2018, 2019) was used to identify protein orthogroups, construct a multiple sequence alignment with MAFFT v7.455 (Katoh and Standley, 2013) and a subsequent maximum likelihood phylogeny with 100 bootstraps constructed with RAxML v8.2.12 (Stamatakis, 2014), compiled with PTHREADS and SSE3, using the Jones-Taylor-Thornton (JTT) model pre- determined by the RAxML automatic amino acid substitution model assignment. Maximum likelihood phylogenies (100 bootstraps) of genera within the family Burkholderiaceae were constructed using RAxML v8.2.12 (Stamatakis, 2014), compiled with PTHREADS and SSE3, using the LT model pre-determined by the RAxML automatic amino acid substitution model assignment. The associated alignments were generated with MAFFT v7.455 (Katoh and Standley, 2013) using protein orthogroups defined by OrthoFinder v2.4.0 (Emms and Kelly, 2018, 2019).

### Calculating Basic Genome Statistics

Genome size and GC content were determined by QUAST v5.0.2 and the average calculated per species within each *Burkholderia* sensu lato genera.

### Predicting and Analyzing Specialized Metabolite Capacity and Diversity

Specialized metabolite BGCs were predicted by antiSMASH v5.1.1 (Blin et al., 2019) and de-replicated by a modified Bacsort cluster_genera.py script (see footnote 1). BGCs tagged as non-ribosomal peptide synthase (NRPS) or polyketide synthetase (PKS) were clustered via cluster_genera.py modified to use Mash v2.2.2 (Ondov et al., 2016) with a *d* = 0.08 distance threshold, while all remaining BGCs were clustered with a *d* = 0.15 distance threshold. BGCs less than 7500 bp in length were removed prior to de-replication. Specialized metabolite BGC capacity was determined per genome by calculating the sum of BGC lengths per genome compared to the genome length. The proportion of distinct BGCs within a species that were shared by other species in the genus was calculated by pooling the distinct BGCs of species within a genus and de-replicating using the same distance thresholds as described above. The de-replicated data was screened with the distinct BGCs of each species to identify any additional representatives within the genus. If the BGC was present in more than one species it was considered shared within the genus.

## Results

### Burkholderiaceae Phylogeny Reveals an Expansion of *Burkholderia* Sensu Lato Genera

To understand the evolutionary context of the seven *Burkholderia* sensu lato genera, an orthogroup-based phylogeny was constructed that consisted of 21 of the 22 validly named genera of the family Burkholderiaceae, in addition to the invalidly published genus “Candidatus *Glomeribacter”* (Figure 1B). No genomic assemblies were available to represent the genus *Thermothrix*. While the seven *Burkholderia* sensu lato genera: *Burkholderia*, *Paraburkholderia*, *Trinickia*, *Caballeronia*, *Mycetohabitans*, *Robbsia*, and *Pararobbisa*, were closely related to each other, they occupied a clade alongside three genera not currently recognized as *Burkholderia* sensu lato: *Chitinasiproducens*, *Mycoavidus*, and “Candidatus *Glomeribacter”* (Figure 1B). *Chitinasproducens* was most closely related to *Robbsia*, while the genera *Mycoavidus*, and Candidatus *Glomeribacter* were closest to the genus *Pararobbsia*. A more comprehensive phylogeny based on 167 genera classified as *Burkholderiaceae* following genome-based taxonomic re-classification by the GTDB (Parks et al., 2020) did not identify any additional genera closely related to the *Burkholderia* sensu lato group beyond those identified previously (Figure 1B).

### Curation and Classification of the *Burkholderia* Sensu Lato Genomic Dataset

Genome assemblies associated with the seven genera of *Burkholderia* sensu lato: *Burkholderia*, *Paraburkholderia*, *Caballeronia*, *Trinickia*, *Mycetohabitans*, *Robbsia*, and *Pararobbsia*, were downloaded from NCBI. Of the 4478 downloaded assemblies, 4166 passed the quality criteria (312 [6.97%] failed) (Supplementary Table 1 and Figure 2). Due to taxonomic significance the genome assembly of type strain *Paraburkholderia steynii* HC1.1ba was included in downstream analyses despite failing the <1000 contig criteria. The kmer-based phylogeny of quality-confirmed representative genomes highlighted 105 genomes that fell outside of the characterized *Burkholderia* sensu lato clades and as a consequence, they were removed from the dataset. Further inspection revealed most of the 105 genomes were unclassified beyond the order Burkholderiales. Interestingly, one genome of a supposed *Burkholderia* strain, *Burkholderia* sp. L27(2015) (GCA_009765705.1), not yet included in the GTDB (March 2021), fell within the *Burkholderia* sensu lato clade, but outside existing genera. The phylogenomic distances to other species were confirmed by inclusion of the genome in the Burkholderiaceae phylogeny, as such, this genome potentially represents a novel genus (Supplementary Figure 1). The final quality-controlled dataset used for subsequent analyses comprised 4062 *Burkholderia* sensu lato genomic assemblies.

Leveraging the taxonomic information of the List of Prokaryotic names with Standing in Nomenclature (LPSN) (Parte et al., 2020) enabled the identification of 139 validly and/or effectively published species’ type strains with associated *Burkholderia* sensu lato genome assemblies. ANI analysis with an initial ANI threshold of 95%, combined with the type strain information, led to the identification of heterotypic synonyms within the 4062-genome collection (Figure 2). Species were considered heterotypic synonyms if the genomes of both type strains possessed ANI values > 97%. Synonyms included “*Paraburkholderia caffeinitolerans*” (Gao et al., 2016) and *“Paraburkholderia dokdonella*” (Jung et al., 2019); with a 99.9% pairwise ANI value, and were subsequently merged into the earlier synonym “*P. caffeinitolerans*” (Figure 2). *Paraburkholderia insulsa* (Dobritsa and Samadpour, 2016) type strain possessed an ANI value of 98.4% compared to *Paraburkholderia fungorum* (Coenye et al., 2001a; Sawana et al., 2014), and was subsequently merged into the earlier synonym *P. fungorum* (Figure 2). *Burkholderia mallei* and *Burkholderia pseudomallei* were exceptions to the synonym merger, despite a pairwise ANI value of 99.3% they were maintained as separate species in the analysis because of their prior taxonomic differentiation due to clinical relevance and disease. Resolution of synonyms resulted in the division of the genome collection into 235 species groups (Figure 2).

In accordance with the GTDB approach (Parks et al., 2020), pairwise ANI values of 95–97% between species group type or proxy-type strains were resolved by increasing their respective ANI circumscription radii to maintain a species distinction by ANI (Figure 2). The only exception to this species group classification was *Burkholderia lata* and two uncharacterized *Burkholderia cepacia* complex species groups, novel_002 and novel_003. Genome assemblies with borderline ANI values (95.0–95.5%) for more than one type or proxy-type strain prevented delineation of these species groups by increasing the ANI species threshold. Consequently, core-gene phylogenomics was required to delineate evolutionary species clades (Supplementary Figure 2). Of the 235 species groups identified, 12 groups (10 validly named and 2 uncharacterized) had their ANI species circumscription radii increased (Figure 2). The inclusion of uncharacterized species groups in the ANI adjustment, rather than merger with named species, was necessary due to the presence of genomes within groups novel_007 and novel_099 that were above and below the 95% threshold for named species but possessed > 95% ANI values to each other.

The 235 species groups were represented by 137 sequenced type strains (129 validly named species and 8 invalidly named species) (Supplementary Table 2) and 98 genomes designated proxy-type strains (Supplementary Table 3) in the absence of sequenced type strains at NCBI for five named species: *Paraburkholderia metrosideri*, *Paraburkholderia unamae*, “Candidatus *Paraburkholderia kirkii*,” and “Candidatus *Paraburkholderia schumanniana*” and 93 uncharacterized species groups (Figure 2). Genomic sequences that could be taxonomically confirmed by comparison to a sequenced type strain of a valid or invalidly named species accounted for approximately 93% of the genomes within the collection (3790 out of 4062). The remaining 272 genomes were represented by the 98 species groups that lacked a sequenced type strain; the majority of which consisted of single-genome species taxa (69 out of 99).

### Taxonomic Diversity of *Burkholderia* Sensu Lato

**FIGURE 3:**
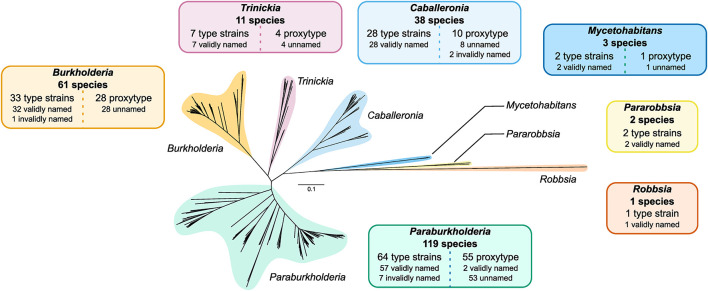
*Burkholderia* sensu lato phylogeny of 235 type strains and proxy type strains. The unrooted phylogeny was based on 731 orthogroups with a minimum of 95.7% of species having single-copy genes in any orthogroup. The seven clades representing the seven genera of the complex are indicated in separate colors. Information boxes indicate the number of species groups represented in each clade, and a break-down of the number of type strains, proxy type strains, validly or invalidly named species, and unnamed/uncharacterized species. Scale bar represents the number of base substitutions per site.

The construction of a phylogeny encompassing the 235 type strains and proxy-type strains permitted a high-resolution insight into the evolutionary taxonomic classification of species within *Burkholderia* sensu lato independent of the existing taxonomic status in public databases (Figure 3 and Supplementary Figure 3). Of the 30610 orthogroups defined in the 235 genomes, the phylogeny was based on an alignment of 731 orthogroups, composed of 207,256 aa positions, with a minimum of 95.7% of species having single-copy genes in any orthogroup (Figure 3). Seven distinct clades were observed in the phylogeny that constituted the seven genera: *Burkholderia*, *Paraburkholderia*, *Caballeronia*, *Trinickia*, *Mycetohabitans*, *Robbsia*, and *Pararobbsia* (Figure 3).

*Burkholderia* and *Paraburkholderia* exhibited the largest expansion in species groups, with both genera approximately doubling their number of known species (Figure 3). An additional 28 uncharacterized species groups were added to the existing 33 named *Burkholderia* species, the majority of which (26 of 28) were localized to the *Burkholderia cepacia* complex (Supplementary Figure 3). Notable expansions included five additional multi-genome species groups closely related to *Burkholderia cenocepacia*: novel_007, novel_008, novel_009, novel_010, and novel_011; and an uncharacterized species, novel_036, within the *Burkholderia pseudomallei* group (Supplementary Figure 3). There was evidence of distinct lineages within the *B. cepacia* complex composed of uncharacterized species groups novel_026 and novel_027 which demarcated the edge of the *B. cepacia* complex (Supplementary Figure 3). *Paraburkholderia* expanded the existing 64 confirmed named species with an additional 55 species groups. As the most populous genus, *Paraburkholderia* also possessed the greatest phylogenomic diversity with evidence of multiple deep-branching lineages (Figure 3). The number of *Trinickia* species increased by four species groups to a total of 11 species. In contrast, only 11 additional species groups were identified across: *Caballeronia* and *Mycetohabitans*, while no uncharacterized species groups were observed in *Robbsia* and *Pararobbsia* (Figure 3).

### Re-Defining Genomes Within Species and Genera Using Phylogenomics

In-depth analysis of the delineated 235 species groups allowed a review of the taxonomic status of all 4062 genomes compared to their current NCBI classification (January 2021). Less than 2% of genomes (61 out of 4062) were re-classified as different genera compared to the NCBI taxonomic status (Supplementary Table 4). Most of these genomes transitioned from *Burkholderia* to the genera *Paraburkholderia* and *Caballeronia* (35 and 19 genomes, respectively), while the remaining genomes were transferred to *Trinickia* and *Mycetohabitans* (five and two genomes, respectively). Important genus re-classifications included the three *Burkholderia* species: *Burkholderia dabaoshanensis*, *Burkholderia novacaledonica*, and *Burkholderia ultramafica*. Both *B. novacaledonica* and *B. ultramafica* were co-characterized and classified within *Burkholderia* (Guentas et al., 2016), however, based on phylogenomics, these species belong to the genera *Caballeronia* and *Paraburkholderia*, respectively (Supplementary Table 4). Recently, both species have been re-classified as *Caballeronia novacaledonica* (Lozano et al., 2021) and *Paraburkholderia ultramafica* (Gao et al., 2021), respectively. While *B. dabaoshanensis* is already recognized as a member of the genus *Trinickia* in the literature (Estrada-de los Santos et al., 2019), NCBI taxonomy still recognized the species as *Burkholderia* during this study. Based on available genomic assemblies, the candidatus species *“Paraburkholderia kirkii* and *“Paraburkholderia schumanniana*” were re-classified as *Caballeronia* species (Supplementary Table 4), however, confirmation of these re-classifications is prevented by the absence of sequenced type strains.

In contrast to the small fraction of genomes that were transferred to a different genus, approximately 11.5% (467 out of 4062) were re-classified as different species compared to the NCBI taxonomy (Supplementary Table 4), excluding eight genomes of named species that lacked sequenced type strains, due to the inability to confirm their original species classification. These eight genomes putatively represented *Paraburkholderia unamae* (novel_106), *Paraburkholderia metrosideri* (novel_107), Candidatus “*Paraburkholderia kirkii*,” Candidatus “*Paraburkholderia schumanniana.*” The putative genomes of both candidatus species were incongruent with their original species grouping, with uncharacterized species “novel_108” representing both “*P. kirkii*” and “*P. schumanniana*,” consequently they could not be assigned to a specific species group. Of the 467 genomes that were re-classified as different species compared to the NCBI taxonomy, 193 were re-classified from one named species to different named species or uncharacterized species group (indicated as novel). The remaining 274 genomes possessed unique taxon ids (unnamed species: sp.) in NCBI and were re-classified under 119 species groups consisting of 45 named species and 74 uncharacterized species groups (Supplementary Table 4).

### Variation in Genome Size and GC Content Across Genera

Following the curation of species groups into genera their average genome sizes and GC content were compared to understand the variation in their basic genomic features. *Paraburkholderia*, *Caballeronia*, and *Burkholderia* species possessed the highest average genomes sizes at 8.35, 7.92, and 7.57 Mbp, respectively (Supplementary Figure 4a). However, these genera also exhibited the largest ranges within *Burkholderia* sensu lato, which encompassed the largest genome, *Paraburkholderia steynii* at 11.45 Mbp, and smallest genome, *Caballeronia* novel_108 at 3.18 Mbp. The smallest average genome sizes were observed in *Mycetohabitans*, 3.55 Mbp (Supplementary Figure 4a). *Burkholderia* species exhibited a high average GC content, 66.73%, and their range did not overlap with other genera, with the exception of two outlier *Paraburkholderia* species (Supplementary Figure 4b). In contrast, considerable overlap in GC content ranges was observed in *Trinickia*, *Pararobbsia*, *Paraburkholderia*, and *Caballeronia*, with genus averages between 62.80 and 63.41%. *Robbsia*, represented by one species, *R. andropogonis*, possessed the lowest genus GC content average at 58.55%, and was only exceeded by the *Paraburkholderia* outlier “*P. bonniea*” (58.72%) (Supplementary Figure 4b). Interestingly, despite the closer phylogenomic relationship between *R. andropogonis* and *Chitinasiproducens palmae*, compared to other genera currently defined in *Burkholderia* sensu lato, the GC content of *C. palmae* JS23 was considerably higher at 66.24%.

### Specialized Metabolite Potential of *Burkholderia* Sensu Lato

In the last decade there has been considerable focus on *Burkholderia* sensu lato as a source of specialized metabolites (Depoorter et al., 2016; Thongkongkaew et al., 2018; Kunakom and Eustáquio, 2019; Mullins et al., 2019; Jones et al., 2021). With our systematic genomic taxonomy platform in place, we sought to understand the genomic potential for specialized metabolite production of each *Burkholderia* sensu lato genus. Prior to analysis, a further quality criterion was applied to reduce the influence of poor-quality genomes on the prediction of specialized metabolite BGCs in the collection. The minimum N50 value was increased from 10 to 80 kbp, resulting in the removal of 412 genomes from the collection, and 11 species groups. These included five named species *Burkholderia catarinensis*, *Paraburkholderia kirstenboschensis*, *Paraburkholderia steynii*, *Caballeronia megalochromosomata*, and *Caballeronia choica*; and six uncharacterized species: *Paraburkholderia* novel_046, novel_061, novel_077, novel_084, and novel 085; and *Caballeronia* novel_108. By increasing the minimum N50 value to 80 kbp the number of genomes within a species that reported a lower-than-average specialized metabolite capacity was reduced compared to the initial minimum 10 kbp N50 value (Supplementary Figure 5).

**FIGURE 4:**
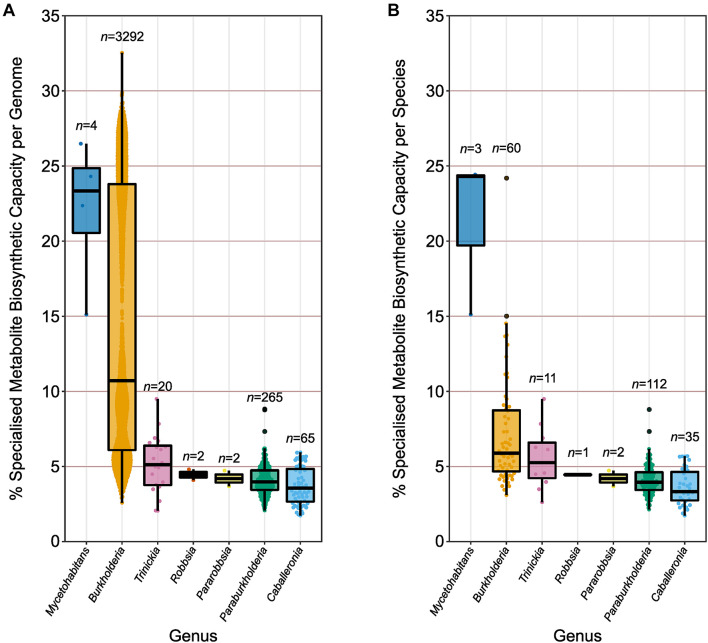
Specialized metabolite biosynthetic gene cluster capacity of individual genomes and species. **(A)** The percentage of sequence per genome (*n* value) predicted to function in specialized metabolite biosynthesis. **(B)** The average percentage of sequence per species (*n* value) predicted to function in specialized metabolite biosynthesis based on genome representatives of the species. Boxplots represent lower quartile, median, and upper quartile. Whiskers represent 1.5x the interquartile range.

Genomes were divided into their respective genera according to the earlier phylogenomic analysis (Figure 3 and Supplementary Figure 3), and their specialized metabolite capacity was calculated using the sum of BGC sequence lengths compared to genome length (Figure 4A). Due to the disparity in the number of genome representatives for different species, the average specialized metabolite BGC capacity was also calculated per species within each genus (Figure 4B). The genus *Mycetohabitans* possessed the highest specialized metabolite capacity within *Burkholderia* sensu lato with a genome mean and median of 22.1 and 23.3%, respectively (Figure 4A). While the genus *Burkholderia* possessed a higher maximum genome specialized metabolite capacity compared to *Mycetohabitans*, the *Burkholderia* mean and median were only 14.6 and 10.7%, respectively. However, *Burkholderia* had the broadest range of capacity for the genera within *Burkholderia* sensu lato, encompassing representatives of all analyzed genera (Figure 4A). The remaining five genera were similar to each other in specialized metabolite capacity with medians between 3.6 and 5.1% (Figure 4A). The same trend was observed when the genome specialized metabolite capacity was averaged per species within their respective genera (Figure 4B). While the mean *Mycetohabitans* species capacity remained the highest across the genera, the mean *Burkholderia* species capacity more than halved (7.2%) compared to the non-averaged species (14.6%). The lower capacity mean was due to the high number of genome representatives in *B. pseudomallei* (*n* = 1400) that were subsequently reduced to a single data point in the species comparison. No species in the remaining five genera possessed over-represented genome numbers, and, as such, the specialized metabolite BGC capacity average per species (Figure 4B) was similar to the non-averaged species comparison (Figure 4A).

### Clade-Restricted Variation in *Burkholderia* Biosynthetic Capacity

**FIGURE 5:**
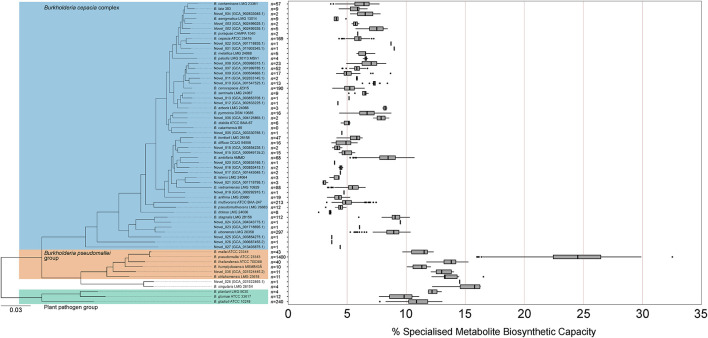
Specialized metabolite biosynthetic gene cluster capacity of *Burkholderia* species. The percentage of genomic sequence per *Burkholderia* species predicted to function in specialized metabolite biosynthesis based on the number of genome representatives of the species (*n* value). The phylogeny was based on 1268 orthogroups with all species having single-copy genes in all orthogroups. The *Burkholderia cepacia* complex, *Burkholderia pseudomallei* group, and plant pathogen group are highlighted by blue, orange, and green boxes, respectively. Boxplots represent lower quartile, median, and upper quartile. Whiskers represent 1.5x the interquartile range.

Given the non-uniform distribution of genome numbers across species in the genus *Burkholderia* (Figure 4A), a species-level analysis was performed to understand the variation in specialized metabolite capacity in relation to phylogenomic relationship (Figure 5). Interestingly, specialized metabolite biosynthetic capacity appeared to be linked to major clades within the genus *Burkholderia* (Figure 5). Species within the plant pathogen group (*B. gladioli*, *B. glumae*, and *B. plantarii*), *B. pseudomallei* group, and sister sub-clade consisting of *Burkholderia singularis* and an uncharacterized species (novel_028) boasted high specialized metabolite capacities (10 of the 11 species above 10%) relative to the *Burkholderia cepacia* complex (Figure 5). Except for certain species, such as *Burkholderia ambifaria*, *Burkholderia ubonensis*, and *Burkholderia stagnalis*, the majority of *B. cepacia* complex species possessed mean (39 out of 49) and median (38 out of 49) specialized metabolite capacities below 7.0% (Figure 5).

### De-Replication of BGCs Ascertains Distinct BGC Count of Species and Genera

**FIGURE 6:**
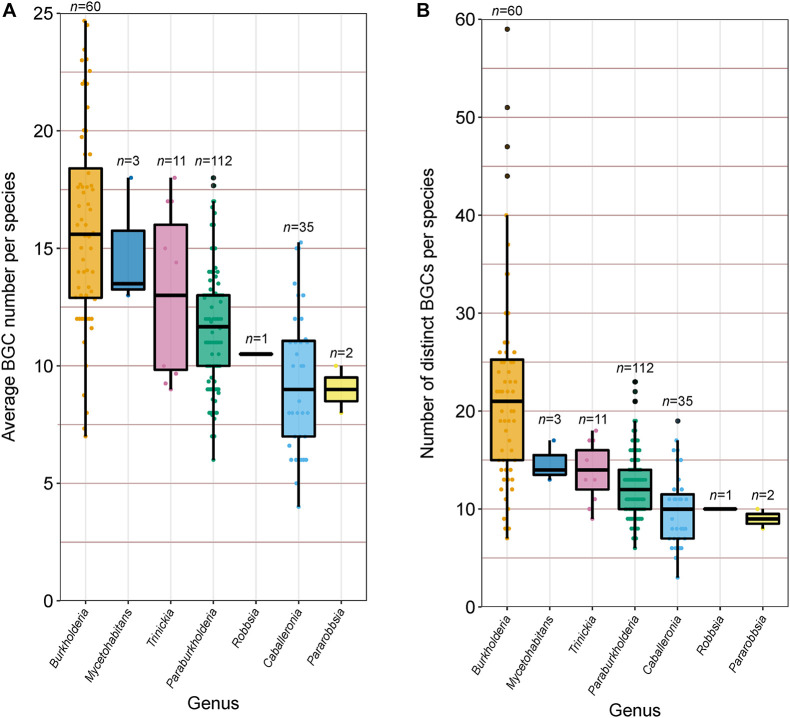
**(A)** Average BGC count per species (*n* value) and **(B)** Number of distinct BGCs harbored by each species (*n* value). Boxplots represent lower quartile, median, and upper quartile. Whiskers represent 1.5x the interquartile range.

In addition to calculating the percentage of each genome predicted to function in specialized metabolite biosynthesis, we calculated the average BGC count per genome within each species group (Figure 6A) and estimated the absolute number of distinct BGCs per species through the de-replication of BGCs shared between genomes (Figure 6B). On average, *Burkholderia* species carried more BGCs than any other genera analyzed with approximately 16 BGC per genome, however, the genus also possessed the greatest range of predicted BGCs at 7–25 BGCs per genome per species (Figure 6A). The genera *Caballeronia* and *Pararobbsia* contained the fewest BGCs per species with an average of nine per genome. At the species level, *B. pseudomallei* and *B. plantarii* averaged close to 25 BGCs per genome, while the single-genome *Caballeronia* species, novel_109, carried only three BGCs, the lowest average BGC count per genome in the collection. Alongside average BGC counts, the number of distinct BGCs per species was estimated to understand the biosynthetic potential of species to produce specialized metabolites across multiple closely related genera (Figure 6B). Similar to the average BGC content per genome, *Burkholderia* species possessed the highest distinct BGC count on average with approximately 22 BGCs, while *Burkholderia ubonensis* harbors up to 59 distinct BGCs. The remaining genera possessed lower distinct BGC counts compared to *Burkholderia* with less than 15 distinct BGCs on average per species, indicative of lower specialized metabolite biosynthetic potential (Figure 6B). However, *Burkholderia* species benefit from high genome representation relative to other genera which bias this result in favor of *Burkholderia*. Of the 224 species groups analyzed for specialized metabolite content, 149 species groups have two or fewer genome representatives. Species groups with limited representation dominated in almost all genera, ranging between 72 and 100% of species per genus, except for *Burkholderia* where limited representative species groups accounted for only 35% of species.

To understand the degree to which BGCs were shared with other species within a genus, the distinct BGCs of species within the most populous genera were pooled by genus, de-replicated, and screened with distinct BGCs per species for evidence of shared BGCs/species specific BGCs. *Burkholderia* species exhibited the highest degree of shared BGCs, with species sharing an average of 76% of BGCs with one or more species. *Caballeronia* and *Paraburkholderia* species shared fewer BGCs with other species within their genera with averages of 55 and 53%, respectively, while *Trinickia* species shared only 5% of their BGCs with other *Trinickia* species. Considerable variation was observed at the species level for *Burkholderia* (11–100%), *Paraburkholderia* (0–100%), and *Caballeronia* (13–100%), compared to *Trinickia* (0–11%). Alongside genuine biological variation in the proportion of shared BGCs, fragmented BGCs artificially inflating distinct BGC counts may also bias these calculations. Indeed, examining the distinct BGCs of *B. gladioli*, which shared only 11% (4 out of 36) of distinct BGCs with other *Burkholderia* species, revealed the presence of multiple BGCs that represent partial sequences of larger BGCs. In contrast, novel_027 and *B. plantarii*, with 13% (1 out of 8) and 31% (8 out of 26) of distinct BGCs shared with other *Burkholderia* species, respectively, had little evidence of fragmented BGCs. Closely related species likely shared BGCs with each other, and thus elevate the degree to which BGCs appear to be shared within a genus, such as *B. cenocepacia*, and the five uncharacterized, but closely related species groups, that each shared between 73% and 93% of distinct BGCs with other *Burkholderia* species.

### Variation in Prevalence of Specialized Metabolite Classes

Performing antiSMASH on a curated collection of over 4000 genomes also afforded an insight into the distribution and prevalence of different predicted classes of specialized metabolite across *Burkholderia* sensu lato. Widespread specialized metabolite BGC classes included those responsible for terpene, phosphonate, and non-ribosomal peptide biosynthesis (Table 1). Terpene BGCs occurred in all 224 species groups investigated for specialized metabolite capacity; while NRPS and phosphonate BGCs were present in a minimum of 88 and 64% of species per genus, respectively (Table 1). Aryl polyene BGCs were also common, occurring in 6 of the 7 genera with a minimum prevalence of 77% of species; all three *Mycetohabitans* species lacked evidence of aryl polyene BGCs (Table 1).

**TABLE 1:** Prevalence of specialized metabolite biosynthetic gene cluster (BGC) classes in species per genera.

		Genus						
		
BGC class	Examples of characterized functions	*Burkholderia*	*Caballeronia*	*Mycetohabitans*	*Paraburkholderia*	*Pararobbsia*	*Robbsia*	*Trinickia*
Homoserine lactone	Quorum sensing	60/60(100%)	7/35(20%)	2/3(67%)	109/112(97%)	1/2(50%)	1/1(100%)	11/11(100%)
Bacterocin	Antibacterial	58/60(97%)	26/35(74%)	1/3(33%)	98/112(88%)	2/2(100%)	0/1(0%)	11/11(100%)
Phosphonate	Antibacterial	59/60(98%)	34/35(97%)	3/3(100%)	100/112(89%)	2/2(100%)	1/1(100%)	7/11(63%)
Lassopeptide	Antibacterial	17/60(28%)	0/35(0%)	3/3(100%)	1/112(1%)	0/2(0%)	0/1(0%)	1/11(9%)
NRPS	Iron chelating, quorum sensing, antibacterial, cytotoxic, biofilm formation, swarming	57/60(95%)	31/35(89%)	3/3(100%)	98/112(88%)	2/2(100%)	1/1(100%)	10/11(91%)
Betalactone	Antimicrobial	45/60(75%)	9/35(26%)	3/3(100%)	43/112(38%)	1/2(50%)	0/1(0%)	2/11(18%)
*trans*AT-PKS	Antibacterial, cytotoxic	15/60(25%)	1/35(3%)	3/3(100%)	2/112(2%)	0/2(0%)	1/1(100%)	0/11(0%)
Terpene	Antibacterial	60/60(100%)	35/35(100%)	3/3(100%)	112/112(100%)	2/2(100%)	1/1(100%)	11/11(100%)
Aryl polyene	Membrane structure/protection from oxidative stress	49/60(82%)	27/35(77%)	0/3(0%)	109/112(97%)	2/2(100%)	1/1(100%)	11/11(100%)

Other specialized metabolite classes exhibited more variation in their prevalence across *Burkholderia* sensu lato. Evidence of homoserine lactone BGCs was widespread in most genera, with BGCs present in all *Burkholderia*, *Robbsia*, and *Trinickia* species, including 97% of *Paraburkholderia* species. In contrast, *Caballeronia* species were markedly lacking in homoserine lactone BGCs with a 20% species representation (Table 1). Lassopeptide BGCs were completely absent in *Caballeronia*, *Pararobbsia*, and *Robbsia*, while *Paraburkholderia* and *Trinickia* each possessed one species with evidence of the lassopeptide metabolite class: *Paraburkholderia mimosarum* and *Trinickia caryophylli*. All three *Mycetohabitans* species and 28% of *Burkholderia* species contained lassopeptide BGCs (Table 1). Surprisingly, of the 224 species groups investigated for specialized metabolites, only 20 species harbored evidence of trans-AT-PKS BGCs. Most of these occurred within *Burkholderia* (15 species), while two species possessed evidence of trans-AT-PKS in both *Paraburkholderia* and *Mycetohabitans*, and one *Caballeronia* species (Table 1).

## Discussion

### High-Resolution Phylogenomics Provides Insights in *Burkholderia* Sensu Lato Taxonomy and Diversity

Maintaining up-to-date taxonomic classifications of bacterial genome assemblies is necessary for facilitating communication of their clinical, industrial, and environmental importance. However, public databases contain multiple examples of erroneous genomic classifications that can bias genomic analyses in the absence of appropriate data curation. Our study exploited over 4000 publicly available *Burkholderia* sensu lato genomic assemblies to assess their diversity and correct their taxonomic status. During review of the study, it became apparent that genome assemblies for the type strains of *Paraburkholderia unamae* and *Paraburkholderia metrosideri* were available at the Integrated Microbial Genomes and Microbiomes database^3^. To aid taxonomic and other research studies done at scale, all researchers should be encouraged to deposit their genome assemblies at databases which allow full public interrogation such as NCBI. We demarcated multiple uncharacterized species groups and their evolutionary relationship to named species, and re-classified hundreds of genome assemblies to their respective species and genera. A limited analysis of the genomic diversity within the *B. cepacia* complex, a sub-group of *Burkholderia* species has also been performed (Jin et al., 2020). Within this analysis, a total of 36 species groups representing 22 named species and 23 uncharacterized species groups were identified based on 116 *B. cepacia* complex genomes (Jin et al., 2020). In comparison, our study delineated the *B. cepacia* complex into 24 named species and 26 uncharacterized genomic species, commensurate to the previous study (Jin et al., 2020). The elevated numbers of both named and uncharacterized species can be explained by the analysis of a significantly larger collection of genomes.

The delineation of genomes into uncharacterized species groups expanded the existing sub-clades within *Burkholderia* sensu lato. For example, an additional five species groups were identified alongside the species clade harboring the *Burkholderia cenocepacia* type strain; several of these species groups were also observed in other genomic studies (Wallner et al., 2019; Jin et al., 2020). Novel group 007 described herein (Figure 2 and Supplementary Figure 3), also known as BCC06 (Jin et al., 2020), was recently the focus of a study to distinguish this *B. cepacia* complex group, representing historical *recA* gene-derived genomovar lineage IIIB, from the closely related *B. cenocepacia* type clade (IIIA) due to observed variations in virulence factor distribution (Wallner et al., 2019). The authors propose the novel, non-validated species name “*Burkholderia servocepacia*” for this genomic grouping within *B. cenocepacia* (Wallner et al., 2019). Our comparison of the “*B. servocepacia*” genomes against our substantive dataset illustrates that an adjustment of the ANI threshold to 96% (Figure 2) is required to fully delineate this genomic species grouping from the *B. cenocepacia* type group. Representatives within both the *B. cenocepacia* and “*B. servocepacia*” species groups are capable of virulent human infections, especially in people with cystic fibrosis, highlighting the need to accurately delineate both species using systematic genomic taxonomy criteria. While the taxonomic split between *B. cenocepacia* and “*B. servocepacia*” remains intact when applied to a larger genome collection (246 and 56 genomes, respectively), the stated distribution of predicted virulence factors as evidence of a potential division in pathogenesis versus environmental adaptation (Wallner et al., 2019) should be viewed with caution. Another group of potential clinical importance was the clustering of 11 genomes that represented a distinct species group, novel_036, in the *Burkholderia pseudomallei* group, alongside the five existing member species (Supplementary Figure 3 and Supplementary Table 4). This represents the latest proposed addition of a species group to the complex since the description of *Burkholderia humptydooensis* (Tuanyok et al., 2017), and may contribute to our understanding of the evolution of this important species complex.

While our study revealed the hidden genomic diversity of the *Burkholderia* sensu lato genera, even further diversity exists based on the multiple named species on the List of Prokaryotic names with Standing in Nomenclature (LPSN) (Parte et al., 2020) that lack genome assemblies. Historically, single-gene or multi-locus sequences were used alongside genetic fingerprinting techniques and biochemical assays to identify strains and species (Coenye et al., 2001b). Subsequent genome sequencing can lead to conflicts in ANI species thresholds where two validly named species possess ANI values greater than 95% (Rusch et al., 2015) or a valid species is actually composed of two closely related (>95%) but distinct lineages (Wallner et al., 2019). *Escherichia* and *Shigella* represent a well-known example of genera that are maintained as distinct for clinical relevance despite genomic relatedness; a distinction also noted for *Bacillus anthracis*/*Bacillus cereus* and *Mycobacterium* species (Ciufo et al., 2018). In contrast, an illustration of the potential genomic diversity being concealed by 16S rRNA gene-based strain identification was the strain *Burkholderia* sp. L27(2015) (GCA_009765705.1) (Lladó et al., 2016), which potentially represents a novel genus within *Burkholderia* sensu lato based on the phylogenomics presented in our study (Supplementary Figure 1). We identified evidence of several distinct lineages within *Paraburkholderia*, the most genetically diverse genus in *Burkholderia* sensu lato based on the comprehensive ortholog-based phylogeny. This sub-clade structure has been observed previously to a limited degree (Beukes et al., 2017; Estrada-de los Santos et al., 2018), however, multiple additional lineages were visible following the inclusion of 119 *Paraburkholderia* type strains and proxy type strains (Figure 3 and Supplementary Figure 3).

The difficulties associated with defining novel bacterial species stem from the historic link between taxonomy and nomenclature (Hugenholtz et al., 2021). Existing rules on bacterial taxonomy require metabolic and physiological data to define a novel species, as outlined in nomenclature journals such as the International Journal of Systematic and Evolutionary Microbiology. Our study delineates bacterial species based on genomic relatedness, providing evidence of novel species groups, but falls short of characterizing these species due to a lack of phenotypic information. There is now growing support for the inclusion of genomic data as a minimum standard for delineating and defining novel species, but also recognition of the importance of sequence data quality and genome authenticity (Chun et al., 2018). In the genomic era, the use of ANI to delineate novel species has been widely adopted by the microbial systematics community (Chun et al., 2018; Ciufo et al., 2018; Parks et al., 2020), with multiple publications converging on a species ANI threshold of 95% or higher (Goris et al., 2007; Richter and Rosselló-Móra, 2009; Jain et al., 2018; Parks et al., 2020). Although the use of public genomic data originating from different sequencing platforms inhibits our ability to confirm sequencing accuracy, many genomic assemblies were deposited during the Illumina era of sequencing, which possesses high accuracy with the exception of long repetitive regions. In addition, while ANI was used to demarcate species boundaries for the initial large scale analysis of our 4000 Burkholderiales genomes, core-gene phylogenomics (Supplementary Figure 2) was ultimately used within closely related species groups where necessary as a high-resolution confirmation of taxonomic delineation.

Despite the considerably expanded genome collection analyzed, the average genus GC content showed similarities to previous studies. The average GC content for *Burkholderia*, *Paraburkholderia*, *Robbsia*, and *Mycetohabitans* was equivalent to previous calculations made prior to the split of these genera from *Burkholderia* (Estrada-de Los Santos et al., 2013). However, our analysis has updated the range in GC content of *Burkholderia* sensu lato compared to previous studies (Dobritsa and Samadpour, 2016; Vandamme et al., 2017; Estrada-de los Santos et al., 2018). While previous publications have provided genome sizes of multiple *Burkholderia* sensu lato species (Beukes et al., 2017; Vandamme et al., 2017) there appears to be no reference of average genome sizes or size ranges for *Burkholderia* sensu lato genera similar to those provided in this analysis.

### Defining the Specialized Metabolite Biosynthetic Capacity of *Burkholderia* Sensu Lato Genera

The natural product capacity of individual *Burkholderia* species has been previously explored to understand BGC distributions and facilitate discovery of uncharacterized metabolites (Mullins et al., 2019; Jones et al., 2021); similar to species of other genera, such as *Bacillus velezensis* (Mullins et al., 2020). However, despite a successful history of natural product discovery in *Burkholderia*, considerably more genome mining analyses have been performed at the broader genus-level in other genera. Large scale multi-species bioinformatic analyses have screened other talented genera including *Bacillus* (Grubbs et al., 2017), *Streptomyces* (Belknap et al., 2020), and *Salinispora* (Letzel et al., 2017), revealing previously uncharacterized BGCs and chemical diversity. The estimation of the number of distinct BGCs of each species compared to the average count highlights the potential strain variation in natural product capacity (Figure 6). Similar trends have been observed in *Streptomyces*, where strains of the same species can vary considerably in both their BGC abundance and class diversity (Belknap et al., 2020). Acknowledging this strain-level variation is important for natural product discovery as assessing the biosynthetic potential of species through genome mining of individual strains will inevitably underestimate specialized metabolite diversity, as shown in *Streptomyces* (Belknap et al., 2020).

The main differences between *Burkholderia* and other closely related genera were the presence BGCs encoding lassopeptides or possessing a trans AT-PKS component (Table 1). Multiple BGCs with a trans AT-PKS component have been characterized in *Burkholderia*, such as enacyloxins (Mahenthiralingam et al., 2011), gladiolin (Song et al., 2017), bongkrekic acid (Moebius et al., 2012), and thailandamide (Nguyen et al., 2008), some of which possess bioactivity as antimicrobials or general toxins. Lassopeptides are a class of ribosomally synthesized and post-translationally modified peptides (RiPPS), of which only a few are characterized, such as capistruin (Knappe et al., 2008) and ubonodin (Cheung-Lee et al., 2020), both of which are reported to have RNA polymerase inhibition activity. Many additional specialized metabolites have been characterized in *Burkholderia* with biological functions involved in swarming, biofilm formation, iron chelation, and quorum sensing (Kunakom and Eustáquio, 2019). Due to the functional diversity of specialized metabolites (Kunakom and Eustáquio, 2019) determining the biological reason for the variations in biosynthetic capacity of *Burkholderia* sensu lato genera represents a challenge to understanding their fundamental ecological purpose in these bacteria. In comparison, several metabolic functions appear to be genus specific, such as the widespread presence of benzoate degradation metabolism in *Paraburkholderia*, and the presence of cysteine and methionine metabolism in *Burkholderia*, but their absence in *Trinickia* (Estrada-de los Santos et al., 2018). The differential presence of these metabolic pathways likely reflects the environmental niche or potential pathogenicity of the representative species of these genera.

## Conclusion

The continuous shifting and refinement of *Burkholderia* sensu lato taxonomy introduces challenges to capturing and defining its current standing in the literature. This study provides a new benchmark of *Burkholderia* sensu lato taxonomy and genomic diversity through genome clustering, ANI analyses, and high-resolution phylogenomics. The resulting taxonomic insights from the analysis of over 4000 genomes permitted us to assess the specialized metabolite biosynthetic capacity of the multi-genus complex. Knowledge of species and genus biosynthetic capacities, and degree of BGC sharing, will hopefully guide targeted exploitation of natural product diversity in *Burkholderia* sensu lato.

## Data Availability Statement

All genome assemblies used during this work are available through the National Center for Biotechnology Information (NCBI) public database. A list of genome assembly accessions and their species group assignments is available in Supplementary Table 4.

## Author Contributions

AM: conceptualization, data curation, formal analysis, investigation, methodology, software, validation, visualization, and writing – original draft. EM: funding acquisition and supervision. AM and EM: project administration, resources, and writing – review and editing. Both authors contributed to the article and approved the submitted version.

## Conflict of Interest

The authors declare that the research was conducted in the absence of any commercial or financial relationships that could be construed as a potential conflict of interest.

## Publisher’s Note

All claims expressed in this article are solely those of the authors and do not necessarily represent those of their affiliated organizations, or those of the publisher, the editors and the reviewers. Any product that may be evaluated in this article, or claim that may be made by its manufacturer, is not guaranteed or endorsed by the publisher.

## Footnotes

1
https://github.com/rrwick/Bacsort

2
https://github.com/kblin/ncbi-genome-download

3
https://img.jgi.doe.gov
