# Supplementary material for: The Hidden Genomic Diversity, Specialized Metabolite Capacity, and Revised Taxonomy of Burkholderia Sensu Lato
Source: Front Microbiol. 2021 Sep 22;12:726847. doi: 10.3389/fmicb.2021.726847 (PMC8506256; doi:10.3389/fmicb.2021.726847)
Supplement: Supplementary file 1 [file Data_Sheet_1.pdf]

Supplementary Information

The Hidden Genomic Diversity, Specialized Metabolite Capacity, and Revised Taxonomy of *Burkholderia* sensu lato

Alex J. Mullins[1,*] and Eshwar Mahenthiralingam[1]

1. Microbiomes, Microbes and Informatics Group, Organisms and Environment Division, School of Biosciences, Cardiff University, Cardiff, United Kingdom

Correspondence: Alex J. Mullins, MullinsA@cardiff.ac.uk

Keywords: *Burkholderia* sensu lato, phylogenomics, taxonomy, average nucleotide identity, biosynthetic gene clusters, specialized metabolites

Contents:

Supplementary Figures

Fig. S1. Burkholderiaceae phylogeny highlighting potentially novel genus.

Fig. S2. Core-gene phylogeny of *Burkholderia lata* and closely related uncharacterised species groups.

Fig. S3. *Burkholderia* sensu lato phylogeny of 235 species groups.

Fig. S4. Comparison of genome sizes and GC content between genera.

Fig. S5. Relationship between genome N50 value and predicted biosynthetic gene cluster content.

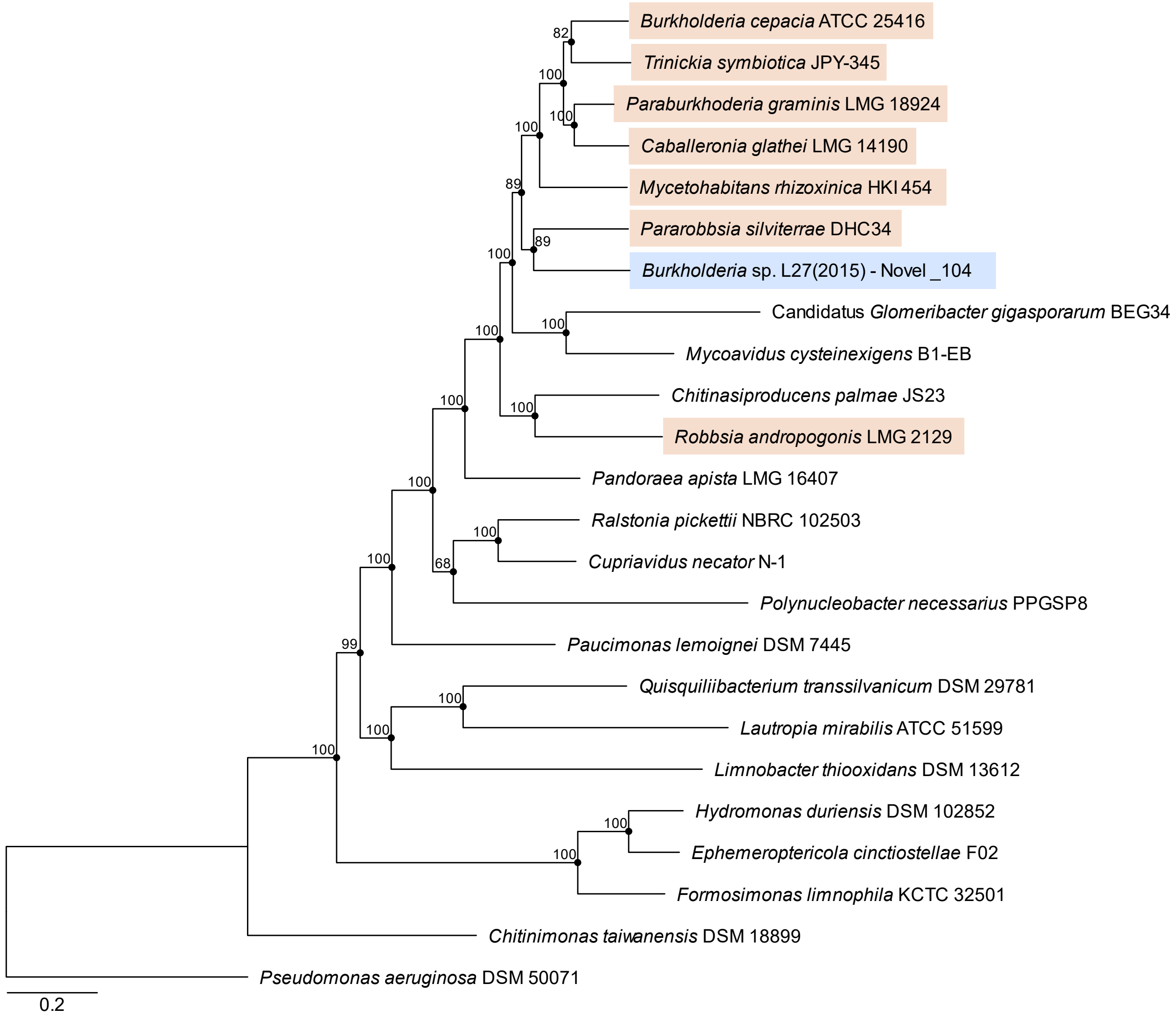

Fig. S1. Burkholderiaceae phylogeny highlighting potentially novel genus. The phylogeny of was based on 624 orthogroups with a minimum of 91.7% of species having single-copy genes in any orthogroup. The maximum likelihood phylogeny was constructed using the LT model determined by the RAxML automatic amino acid substitution model assignment with 100 bootstraps. The phylogeny contains type strains of 21 of the 22 validly named species, a representative of the invalidly named "Candidatus *Glomeribacter*" genus, and the potentially novel genus representative, novel_104. The phylogeny was rooted with *Pseudomonas aeruginosa* DSM 50071. Genera currently recognised as *Burkholderia* sensu lato group are highlighted in orange, while the potentially novel genus representative is shown in blue. Scale bar represents the number of base substitutions per site.

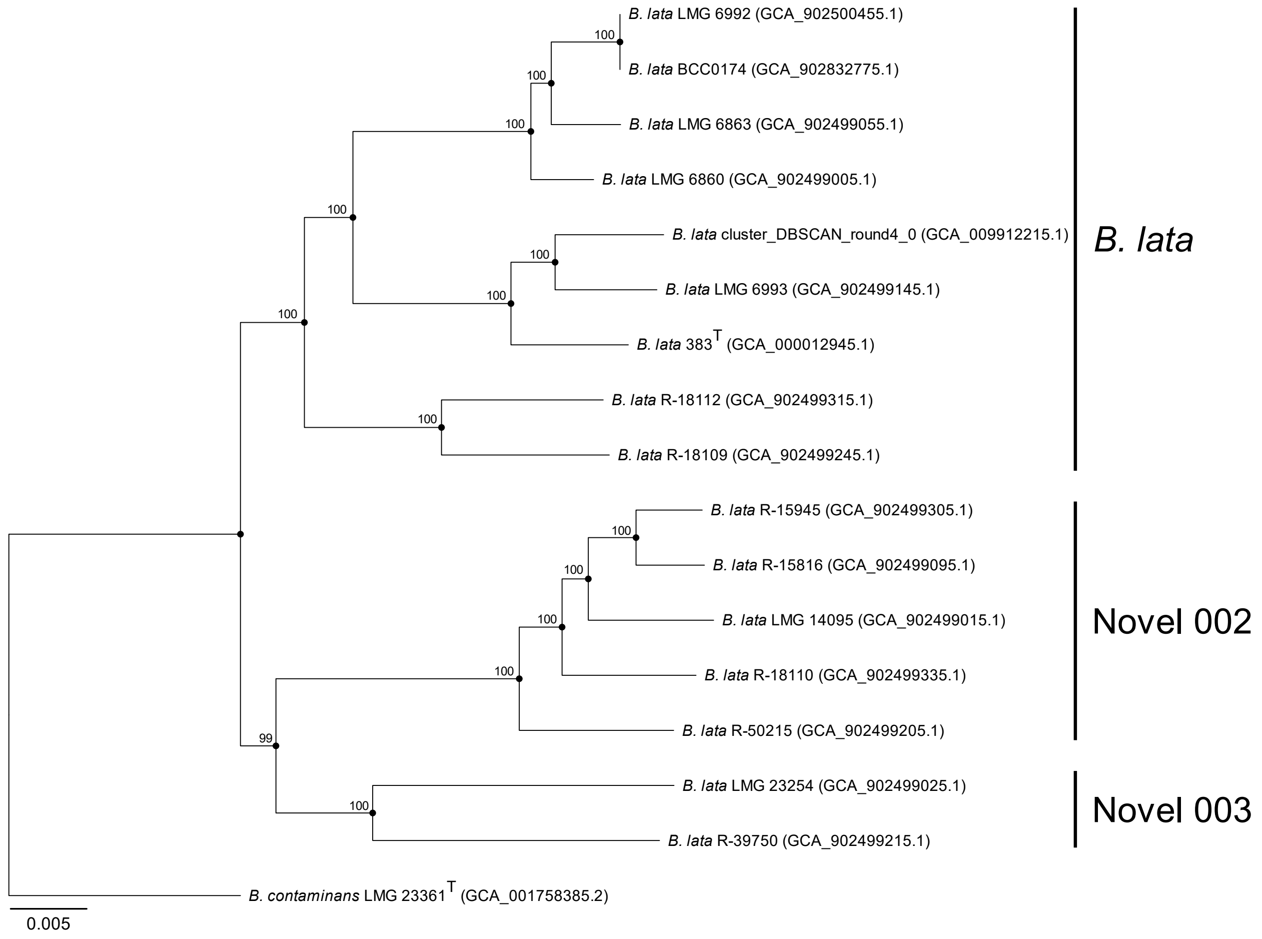

Fig. S2. Core-gene phylogeny of *Burkholderia lata* and closely related uncharacterised species groups. Phylogenomics of these closely related genomes identified three clades that could not be resolved with ANI analyses alone. The core-gene alignment was composed of 3975 genes identified in >99% of genomes. The maximum likelihood phylogeny was constructed with a general time reversible model and gamma model of rate heterogeneity with 100 bootstraps. The phylogeny was rooted with *Burkholderia contaminans* LMG 23361. Scale bar represents the number of base substitutions per site.

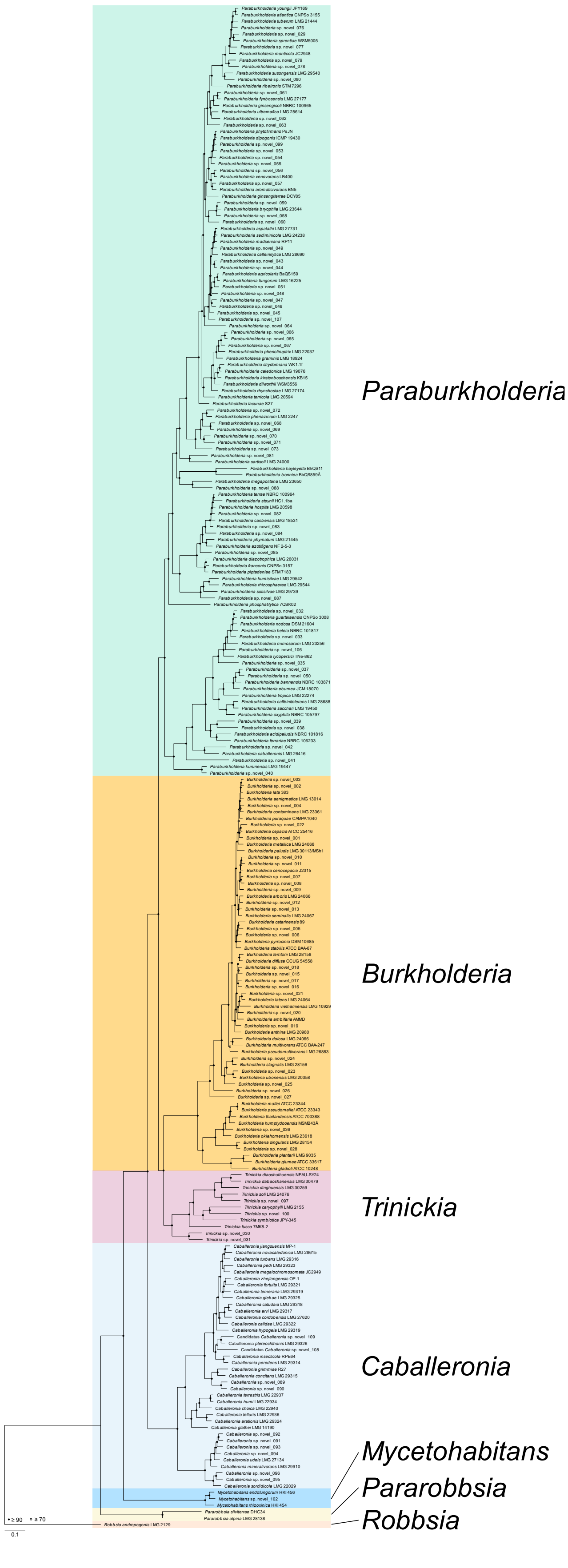

Fig. S3. *Burkholderia* sensu lato phylogeny of 235 species groups. Phylogeny based on the type strains and proxy type strains of the 235 species groups of named and uncharacterised species, respectively. Genera boundaries are indicated with coloured boxes. The phylogeny was based on 731 orthogroups with a minimum of 95.7% of species having single-copy genes in any orthogroup. Scale bar represents the number of base substitutions per site. Solid node circles indicate bootstrap values ≥90, and hollow node circles indicate bootstrap values ≥70. The same phylogeny is presented in Fig. 3.

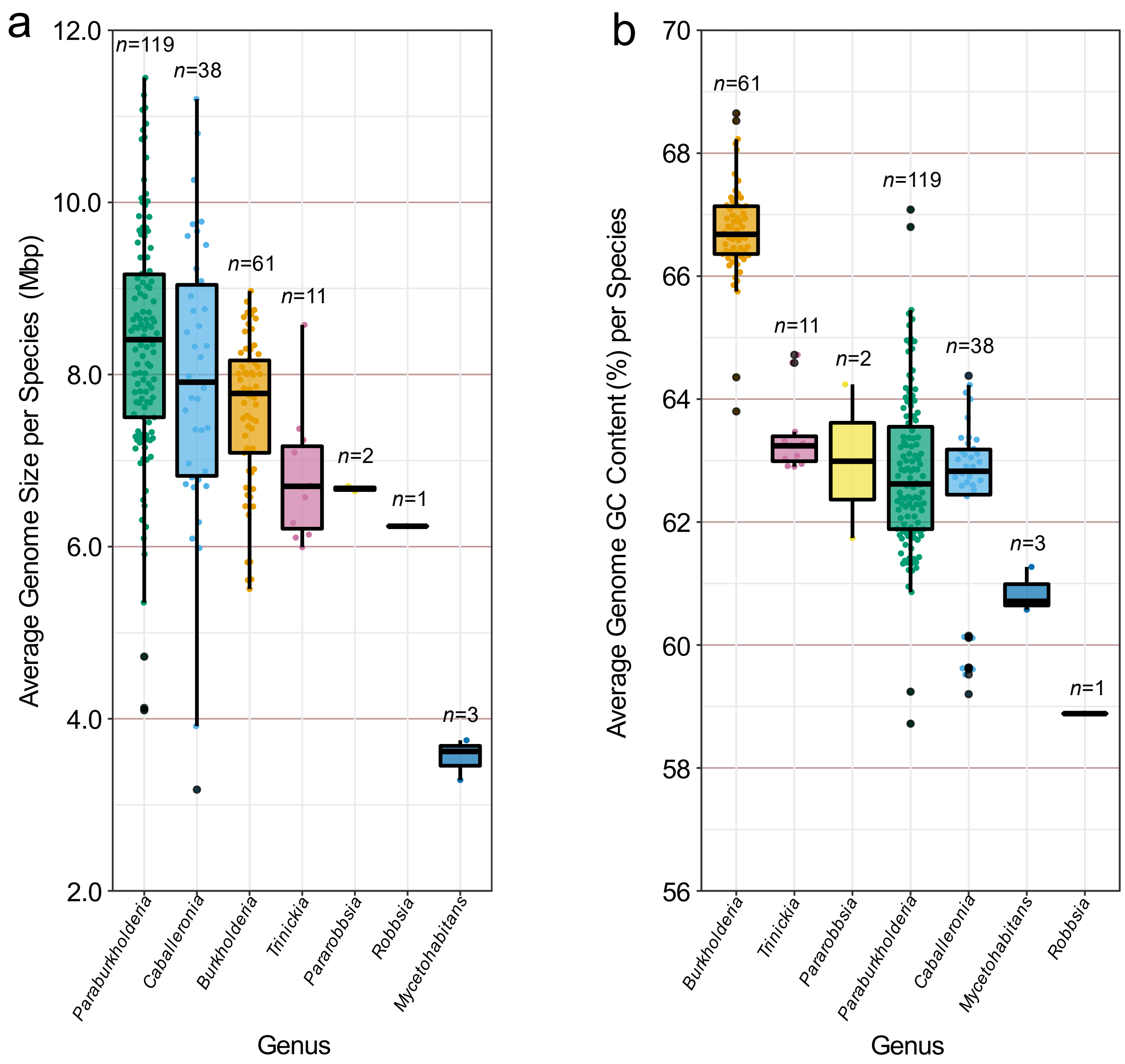

Fig. S4. Comparison of genome sizes and GC content between *Burkholderia* sensu lato genera. The average genome size and GC content was calculated per species group and displayed per genera. Boxplots represent lower quartile, median, and upper quartile. Whiskers represent 1.5x the interquartile range.

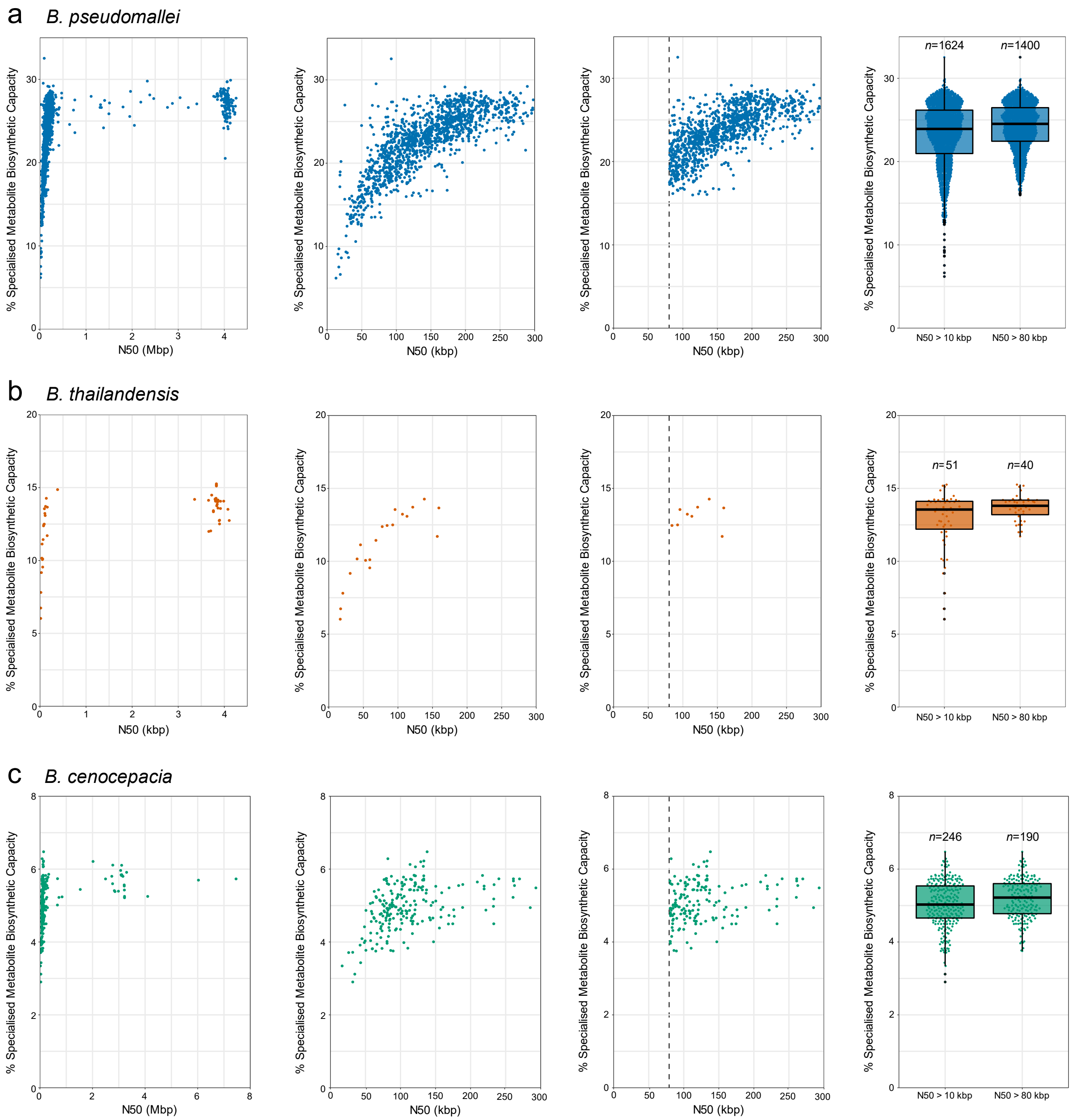

Fig. S5. Relationship between genome N50 value and predicted biosynthetic gene cluster content. (a) *Burkholderia pseudomallei* genomes; **(b)** *Burkholderia thailandensis* genomes; **(c)** *Burkholderia cenocepacia* genomes. Column 1 shows the relationship between % specialised metabolite biosynthetic capacity per genome and N50 value greater than 10 kbp. Column 2 shows the same relationship but focusses on N50 values up to 300 kbp. Column 3 displays the same graph column 2 but excludes genomes possessing N50 values below 80 kbp. Column 4 compares the specialised metabolite biosynthetic capacity before and after the exclusion of genomes with N50 values below 80 kbp.
